# Development and testing of a random forest-based machine learning model for predicting events among breast cancer patients with a poor response to neoadjuvant chemotherapy

**DOI:** 10.1186/s40001-023-01361-7

**Published:** 2023-09-30

**Authors:** Yudi Jin, Ailin Lan, Yuran Dai, Linshan Jiang, Shengchun Liu

**Affiliations:** 1 Department of Breast and Thyroid Surgery, The First Affiliated Hospital of Chongqing Medical University, Chongqing, 400016 China; 2 Department of Pathology, Chongqing Key Laboratory for Intelligent Oncology in Breast Cancer (iCQBC), Chongqing University Cancer Hospital, Chongqing, 400030 China

**Keywords:** Breast cancer, Machine learning, Random forest, Logistic regression, Event

## Abstract

**Background:**

Breast cancer (BC) is the most common malignant tumor around the world. Timely detection of the tumor progression after treatment could improve the survival outcome of patients. This study aimed to develop machine learning models to predict events (defined as either (1) the first tumor relapse locally, regionally, or distantly; (2) a diagnosis of secondary malignant tumor; or (3) death because of any reason.) in BC patients post-treatment.

**Methods:**

The patients with the response of stable disease (SD) and progressive disease (PD) after neoadjuvant chemotherapy (NAC) were selected. The clinicopathological features and the survival data were recorded in 1 year and 5 years, respectively. Patients were randomly divided into the training set and test set in the ratio of 8:2. A random forest (RF) and a logistic regression were established in both of 1-year cohort and the 5-year cohort. The performance was compared between the two models. The models were validated using data from the Surveillance, Epidemiology, and End Results (SEER) database.

**Results:**

A total of 315 patients were included. In the 1-year cohort, 197 patients were divided into a training set while 87 were into a test set. The specificity, sensitivity, and AUC were 0.800, 0.833, and 0.810 in the RF model. And 0.520, 0.833, and 0.653 of the logistic regression. In the 5-year cohort, 132 patients were divided into the training set while 33 were into the test set. The specificity, sensitivity, and AUC were 0.882, 0.750, and 0.829 in the RF model. And 0.882, 0.688, and 0.752 of the logistic regression. In the external validation set, of the RF model, the specificity, sensitivity, and AUC were 0.765, 0.812, and 0.779. Of the logistics regression model, the specificity, sensitivity, and AUC were 0.833, 0.376, and 0.619.

**Conclusion:**

The RF model has a good performance in predicting events among BC patients with SD and PD post-NAC. It may be beneficial to BC patients, assisting in detecting tumor recurrence.

**Supplementary Information:**

The online version contains supplementary material available at 10.1186/s40001-023-01361-7.

## Introduction

In recent years, the incidence of breast cancer (BC) has increased dramatically and has surpassed lung cancer as the most common malignant tumor worldwide, with an estimated of over 2 million new BC cases annually [1]. However, with the amelioration of the treatments, the prognosis of BC patients has improved largely during the past few decades, with an over 90% overall survival [2].

Neoadjuvant chemotherapy (NAC), which is widely used currently, aims to downstage the advanced BC, change inoperable tumors to operable, and allow the performance of breast-conserving surgery [3]. Patients with pathological complete response (pCR) generally have a better long-term prognosis [4, 5]. It was indicated that the response rate was significantly associated with tumor size, lymph node status, hormone receptor (HR), human epidermal growth factor receptor 2 (HER2), Ki67, and P53 [6–8]. Nevertheless, quite a few patients showed residual tumors, especially stable or progressed tumors after receiving NAC. Previous research has pointed out that poor response to NAC was significantly associated with tumor recurrence and mortality [9]. Early detection and timely treatment may prevent damage.

Researchers have tried to predict the prognosis of tumor patients [10–15], and the most commonly used method for developing models was Cox proportional hazard regression [16]. However, the proportional hazard assumption was not always effective. Additionally, Yu et al. introduced innovative individualized clinical decision nomograms, which based on the application of MRI-based machine learning that can accurately predict lymph node status and DFS [17]. Massafra et al. have conducted ensemble machine learning approach. By combining the predictions of three baseline models through a voting mechanism and utilizing a grid search procedure, their method successfully predicts the occurrence of invasive disease events at 5- and 10-year intervals in breast cancer patients [18]. Mikhailova et al. applied six types of machine learning models and confirmed the feasibility of machine learning in predicting breast cancer recurrence [19]. The performance of the models has always been a subject of controversy.

Random forest (RF) is a kind of machine learning algorithm, that uses multiple decision trees to identify, classify, and predict target data [20]. For example, an RF model could be used for predicting decreased quality of life in thyroid cancer patients after thyroidectomy [21]. It has been confirmed that RF could reach satisfying sensitivity and specificity. Therefore, in this study, we aimed to construct and test a machine learning model based on the RF algorithm, to better classify and predict the event (defined as either (1) the first tumor relapse locally, regionally, or distantly; (2) a diagnosis of secondary malignant tumor; or (3) death because of any reason.) of BC patients after receiving NAC.

## Methods

### Patients selected and study designed

**Fig. 1:**
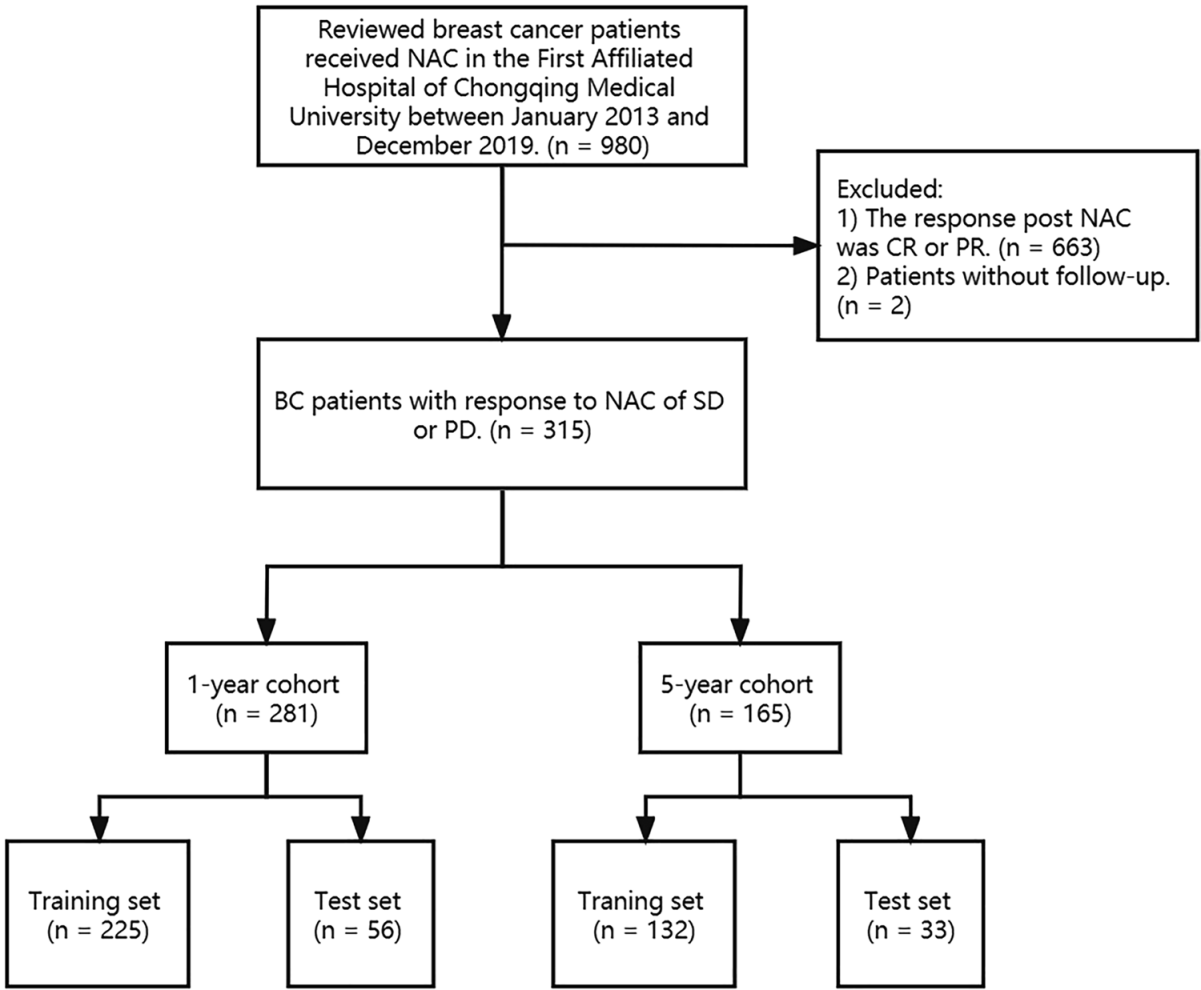
The flowchart of this study

The patients diagnosed as BC in the First Affiliated Hospital of Chongqing Medical University from January 2013 to December 2019 were fully reviewed. This study was approved by the ethics committee of the First Affiliated Hospital of Chongqing Medical University (ID: No. 2020–59). Patients submitted to NAC were selected for the following analysis. According to the Recist guidelines, version 1.1, the response to the NAC included (1) complete response (CR, the tumor disappeared totally by imaging examination and lasted for at least 4 weeks), (2) partial response (PR, decrease no less than 30% in the sum of lesions and last for at least 4 weeks), progressive disease (PD, more than 20% increase in the sum of diameters of lesions), stable disease (SD, neither met the criteria of PR nor did PD) [22, 23]. In this study, patients with SD or PD were included. Of these patients, we first recorded their clinicopathological features, as well as their physical condition post-surgery in 1 and 5 years, respectively. The patients with tumor recurrence or death were divided into the event group, otherwise were into the non-event group. Then, we compared the clinicopathological characteristics between the two groups. Next, based on the Cox regression in both univariate and multivariate analysis, we screened out the variables for constructing the random forest model. For comparison, a logistic model was developed. Finally, we validated the performance of the model. Figure 1 displays the study process.

### Treatment protocol

NAC was performed according to the guidelines of the Chinese Society of Clinical Oncology (CSCO). The CSCO has a longstanding dedication to conducting Continuing Medical Education and facilitating multi-center collaborative research in clinical oncology. Their efforts aim to promote the standardization of tumor diagnosis and treatment, ultimately enhancing the academic excellence of clinical oncology in China. If the patients met the requirement, they would be submitted to the NAC in a week. The treatment protocol was TEC (docetaxel 75 mg/m^2^, epirubicin 75 mg/m^2^, and cyclophosphamide 500 mg/m^2^) or EC (epirubicin 75 mg/m^2^, and cyclophosphamide 500 mg/m^2^), drugs were administered every 21 days. After 4–6 cycles of NAC, the response was evaluated by both the clinicians and the pathologists [24]. Mastectomy or breast-conserving surgery plus axillary lymphadenectomy was performed subsequently. Furthermore, the systemic therapy procedures were as follows: First, patients with an IHC core of 3 + or 2 + with positive FISH was defined as HER2-positive need receive anti-HER2 targeted therapy. Second, patients with estrogen receptor positive or progesterone receptor positive require hormone therapy. Third, the indications of chemotherapy included: (1) high level of Ki67 index; (2) triple negative breast cancer; (3) HER2 positive; (3) regional lymph node positive; (4) Histological grade III or higher; (5) Genetic testing indicates a high risk of recurrence; (6) a relatively larger tumor size. Fourth, for node-positive disease, all the patients receive PMRT to the chest wall. For the node-negative, tumor less than or equal to 5 cm, and clear margins (≥ 1 mm), PMRT is generally not needed. However, if patients with high-risk, including (1) central tumors; (2) T3; (3) tumors not less than 2 cm with fewer than 10 axillary nodes removed; (4) at least one of (a) grade 3, (b) ER-negative, or (c) lymphovascular invasion, PMRT should be considered [25, 26].

### Pathological evaluation

The immunohistochemistry (IHC) index was assessed based on the core needle biopsy of the tumor. Hormone receptor (HR) positive was defined as estrogen receptor (ER) or progesterone receptors (PR) expressing cells percentage > 1% by IHC. HER2 status was classified according to the IHC score and the result of fluorescence in situ hybridization (FISH). A score of 0 was HER2 negative, a score of 1 + or 2 + without amplification of ERBB2 gene was HER2-low expression, and a score of 3 + or 2 + with ERBB2 gene amplified was HER2 positive. A cut-off value of 1% was used for defining the P53 positive expression [27, 28]. These were evaluated by two pathologists independently and blindly.

### Follow-up

All patients were interviewed by telephone from discharge to February 1st, 2021. Disease-free survival (DFS) was selected for assessing patients’ prognoses. DFS was defined as the period between surgery to the event that first recorded tumor relapse or metastasis. The patients with follow-up time longer than 1 year, or who had an event within 1 year were divided into the 1-year cohort. The patients with follow-up time longer than 5 years, or who had an event within 5 years were divided into the 5-year cohort.

### The construction of RF and logistic regression model

In this study, we first established an RF model in the 1-year cohort, and the outcome variable was whether the patients had an event (defined as tumor relapse or metastasis). Based on the result of the chi-square test and the Cox regression, multiple factors that might influence the outcomes were chosen as explanatory variables, which were used to classify and predict the outcome variables. The data were randomly and independently divided into the training set and the test set according to the ratio of 8:2.

Then, we used the training set for developing optimal models. The importance of each explanatory variable was assessed by evaluating the Mean decrease accuracy and the Mean decrease gini. The higher the value, which means the more important the explanatory variable in the model. The RF model was tested in the test set, and the receiver operating characteristic (ROC) curve and the area under the curve (AUC) were used to measure the accuracy of the models.

Next, the same explanatory variables in the RF model were used to develop a logistic regression to classify and predict the outcome variable in the 1-year cohort. Finally, with the same method as the above mentioned, an RF model and a logistic regression were developed in the 5-year cohort. The performance was compared between the RF model and the logistic regression based on specificity, sensitivity, and AUC.

### Model validation

We screened patients from the Surveillance, Epidemiology, and End Results (SEER) research data, 8 Registries, Nov 2022 Sub (1975–2022). The breast cancer patients—who were stated as no response to NAC and had comprehensive data—were selected. These data were used to validate the previously RF model and logistic regression model which constructed based on the 5-year cohort.

### Statistical analysis

We used IBM SPSS 23.0 (Chicago, IL, USA) and RStudio 1.1.456 (R version 4.2.1) software for statistical analysis. All variables were converted into categorical variables and were described as absolute values. The chi-square test was used to compare categorical data. Cox proportional hazard model was performed to identify prognostic factors in both the univariate analysis and the multivariate analysis. The “randomForest” Package was used for developing the RF model. The “glm” function was used to develop the logistic regression. The “pROC” and “ROCR” packages were used to perform the ROC and evaluate the AUC. In this study, only when *p*-value < 0.05 was considered a significant difference.

## Results

### Patients’ characteristics

A total of 980 BC patients received NAC. Of them, 317 patients were with no response to NAC (297 of SD and 20 of PD). The mean age was 49.3 years old (ranging from 20 to 72). 2 patients refused to receive follow-up. Of the rest 315 patients, the mean follow-up time was 40.4 months (range from 2 to 101). Totally, 82 cases had an event during the follow-up time. 251 patients were with a follow-up time of more than 1 year, and 30 patients had an event within 1 year. Stage T (*P* = 0.017) and N (*P* = 0.031), HR (*P* = 0.046) and HER2 (*P* = 0.013) were significant with the event. 165 patients underwent follow-up for more than 5 years and 80 patients had an event within 5 years. HR and HER2 were significant in the 5-year group (Table 1). The Cox regression revealed that stage T, HR, HER2, and P53 were risk predictors for DFS in the univariate analysis, while HR, HER2, and P53 were independent predictive factors in the multivariate analysis (Table 2).

**Table 1:** Clinicopathological features of patients in 1-year cohort and 5-year cohort

	1-year cohort			5-year cohort		
	Event	Non-event	*P*-value	Event	Non-event	*P*-value
Age			0.555			0.707
≤ 40	7	40		13	18	
40–60	19	174		56	56	
> 60	4	37		11	11	
Histological type			0.111			0.237
Invasive ductal tumor	26	237		72	81	
Others	4	14		8	4	
T			0.017			0.064
T1 + T2	20	214		61	75	
T3	10	37		19	10	
N			0.031			1
N0 + N1	19	204		59	63	
N2 + N3	11	47		21	22	
HR			0.046			0.001
Negative	17	92		45	25	
Positive	13	159		35	60	
HER2			0.013			< 0.001
Negative	5	34		12	10	
Low expression	10	150		34	62	
Positive	15	67		34	13	
Ki67			0.718			0.225
≤ 20	16	150		42	56	
20–30	8	52		19	15	
> 30	6	49		19	14	
P53			0.103			0.228
Negative	6	91		19	28	
Positive	24	160		61	57

**Table 2:** Cox regression of the patients’ cohort using univariate and multivariate analysis

	Univariate analysis			Multivariate analysis		
	*P*-value	Hazard ratio	95% CI	*P*-value	Hazard ratio	95% CI
Age (≤ 60 vs. > 60)	0.795	1.088	0.576–2.054			
Histological type (Invasive tumor vs. other type)	0.083	0.524	0.253–1.088			
T (T1 + T2 vs. T3)	0.048	0.595	0.355–0.995	0.138	0.817	0.626–1.067
N (N0 + N1 vs. N2 + N3)	0.167	0.704	0.428–1.158			
HR (negative vs. positive)	< 0.001	2.298	1.485–3.557	0.008	1.373	1.087–1.733
HER2				0.024		
Negative vs. positive	0.248	0.678	0.351–1.31	0.67	0.864	0.44–1.694
Llow expression vs. positive	< 0.001	0.427	0.266–0.687	0.008	0.504	0.304–0.835
Ki67 (≤ 20 vs. > 20)	0.162	0.733	0.475–1.133			
P53 (negative vs. positive)	0.049	0.602	0.363–0.998	0.025	0.743	0.574–0.963

### Development of RF model and logistic regression

**Fig. 2:**
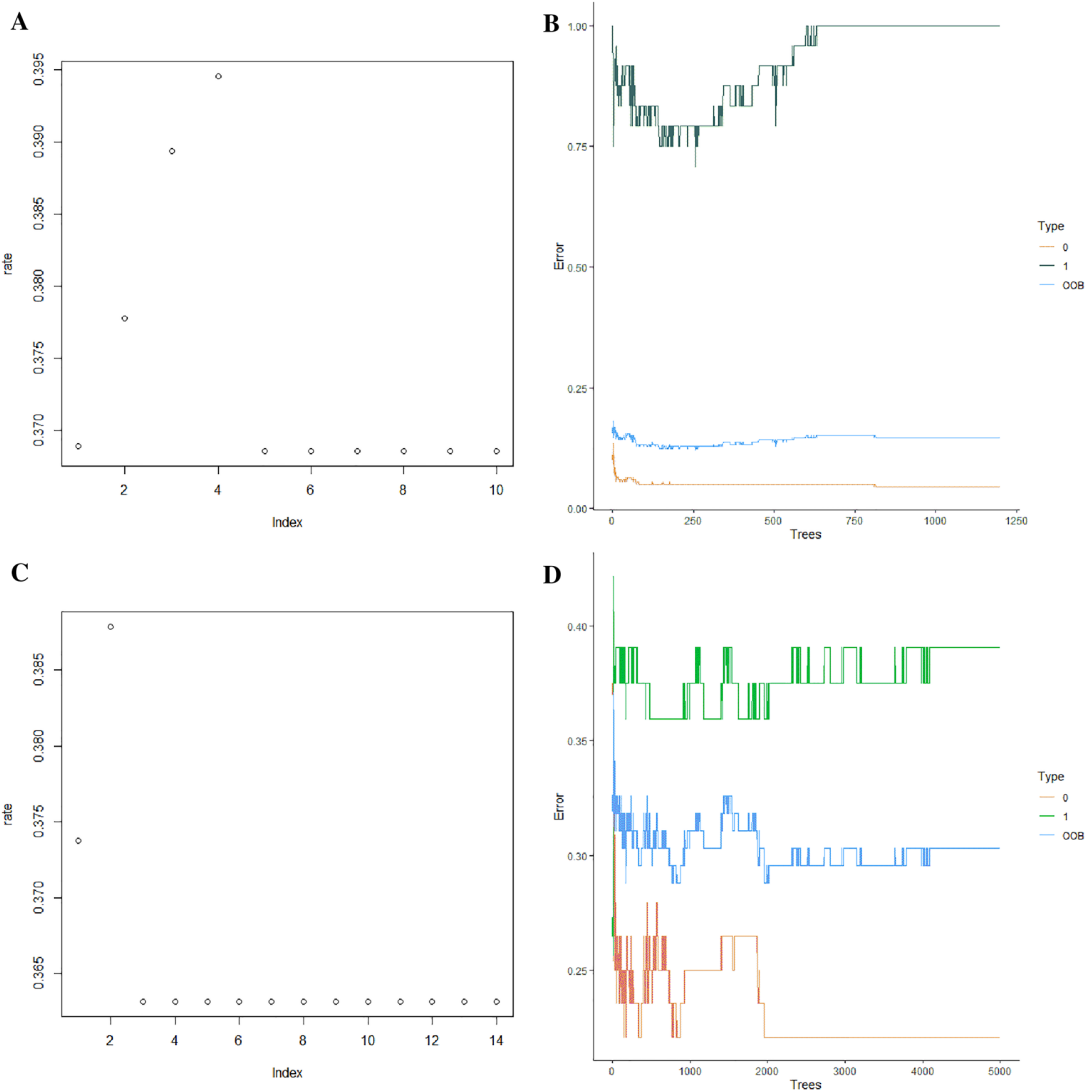
The choice of the number of mtry and ntree in the 1-year cohort (**A**, **B**) and the 5-year cohort (**C**, **D**), respectively. “0” means the error rate of predicting the probability of non-event. “1” means the error rate of predicting the probability of the event. “OOB” means the error rate of out-of-bag

**Fig. 3:**
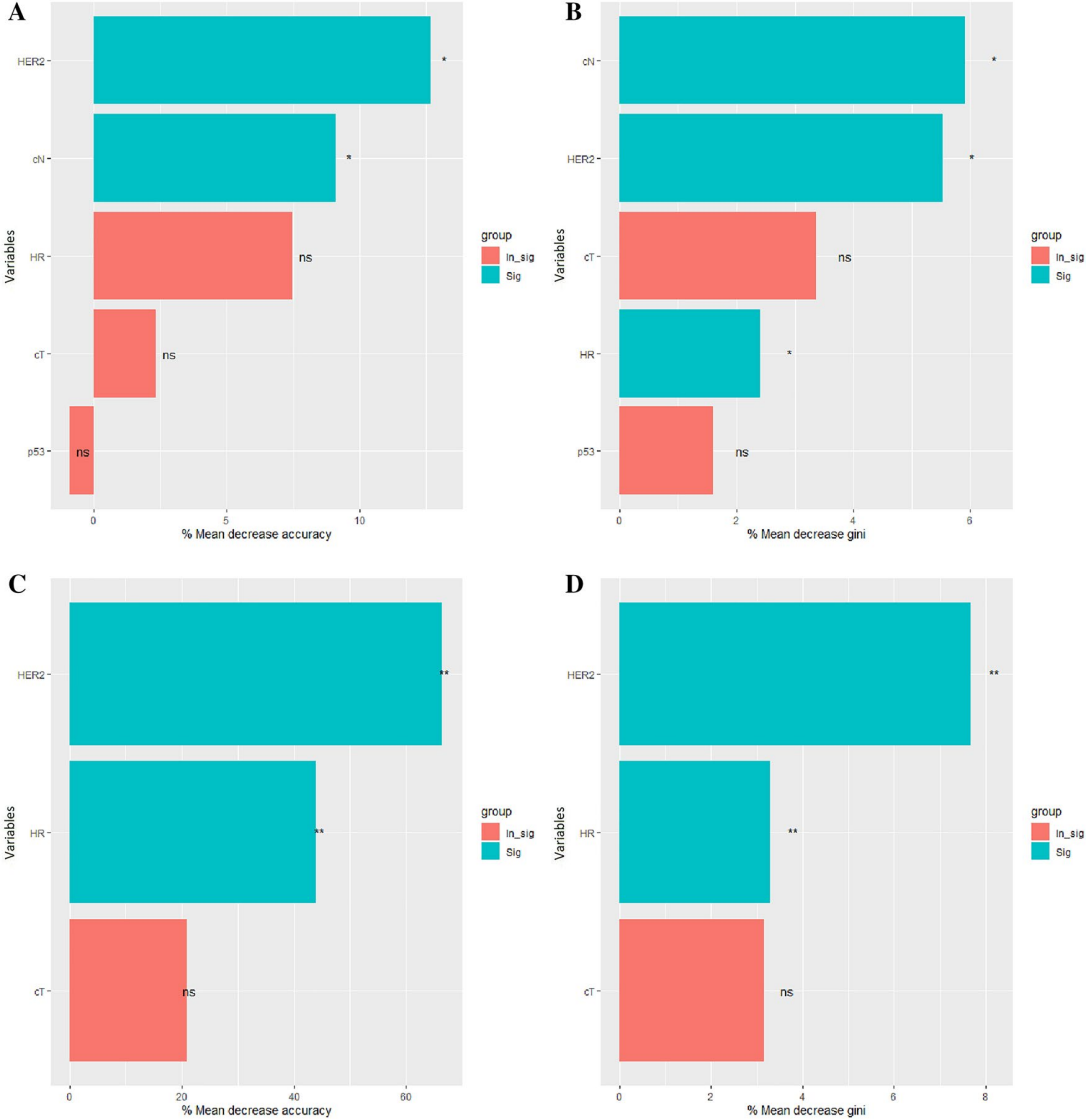
The mean decrease accuracy and mean decrease gini index in the 1-year cohort (**A**, **B**) and the 5-year cohort (**C**, **D**), respectively. Both the “In_sig” and “ns” means not significant. “Sig” means significant. “*” means *P*-value < 0.05. “**” means *P*-value < 0.01

Based on the R package “randomForest”, RF models for predicting the probability of the event in the 1-year cohort and 5-year cohort were developed, respectively. Firstly, in the 1-year cohort, 225 patients were divided into the training set while 56 were into the test set. After measurements, Stage T, stage N, HR, HER2, and P53 were selected as explanatory variables. When mtry = 5 (Fig. 2A), ntree = 1200 (Fig. 2B), the model would have the lowest error. The model was significant at *P* = 0.01 and with an out-of-bag (OOB) error of 14.67%. Figure 3A, B shows the mean decrease accuracy and mean decrease gini index of each variable.

**Fig. 4:**
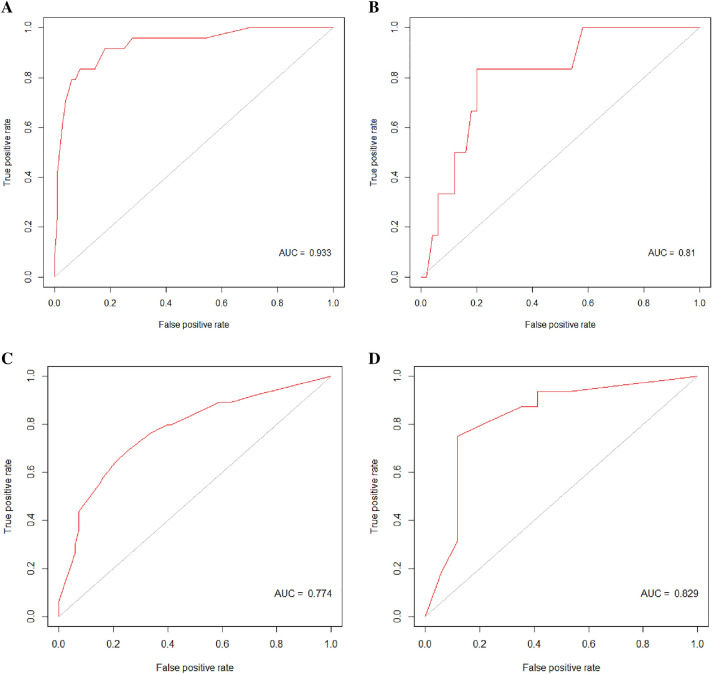
The ROC curve of the random forest model: training set and test set of the 1-year cohort (**A**, **B**); training set and test set of the 5-year cohort (**C**, **D**)

**Fig. 5:**
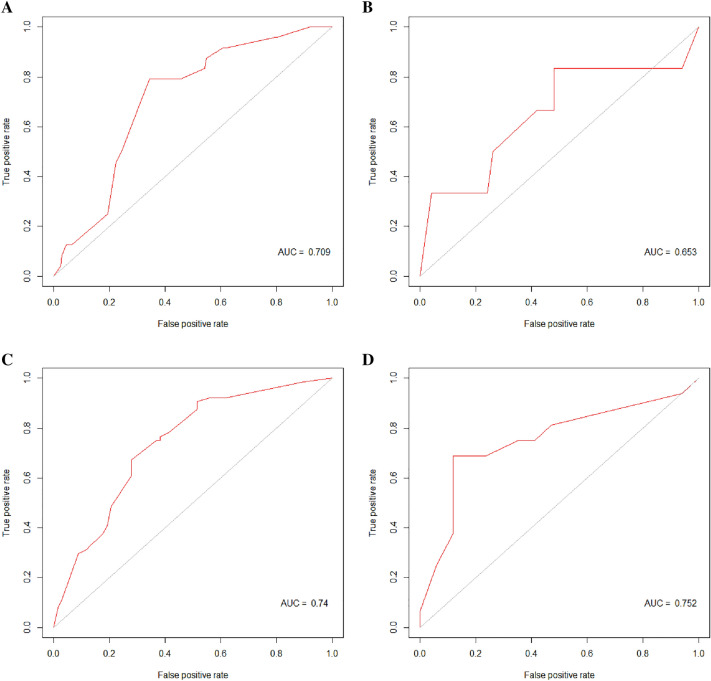
The ROC curve of the logistic regression: training set and test set of the 1-year cohort (**A**, **B**); training set and test set of the 5-year cohort (**C**, **D**)

Secondly, the model was tested in the test set. The ROC of the RF model was performed and the specificity, sensitivity, and AUC were 0.800 (95% CI was 0.659–0.895), 0.833 (95% CI was 0.365–0.991), and 0.810 (95% CI was 0.640–0.980, Fig. 4B) in the test set, respectively. The positive predictive value (PPV), negative predictive value (NPV) and F1 score were 0.333, 0.976 and 0.476, respectively. Thirdly, logistic regression was conducted with the same explanatory variables. The specificity, sensitivity, and AUC were 0.520 (95% CI was 0.376–0.661), 0.833 (95% CI was 0.365–0.991), and 0.653 (95% CI was 0.363–0.944, Fig. 5B). The PPV, NPV and F1 score were 0.172, 0.963 and 0.285 respectively.

Next, we used the same method to establish the RF model and logistic regression in the 5-year cohort, 132 patients were divided into the training set while 33 were into the test set. cT, HR, and HER2 were selected as explanatory variables. Figure 3C, D shows the mean decrease accuracy and mean decrease gini index of each variable. Here in the RF model, we chose mtry = 3 (Fig. 2C) and ntree = 4500 (Fig. 2D). The RF model was significant at *P* < 0.01 and with an OOB error of 30.30%. The specificity, sensitivity, and AUC were 0.882 (95% CI was 0.623–0.979), 0.750 (95% CI was 0.474–0.917), and 0.829 (95% CI was 0.676–0.982) in the test set (Fig. 4D). The PPV, NPV and F1 score were 0.857, 0.789 and 0.80, respectively. Of the logistic regression, the specificity, sensitivity, and AUC were 0.882 (95% CI was 0.623–0.979), 0.688 (95% CI was 0.415–0.879), and 0.752 (95% CI was 0.575–0.829) in the test set (Fig. 5D). The PPV, NPV and F1 score were 0.846, 0.75 and 0.759 respectively. Relative information is displayed in Figs. 2, 5 and Table 3.

**Table 3:** The specificity, sensitivity, AUC, PPV, NPV and F1 score of the train set and test set in 1-year cohort and 5-year cohort, respectively

		1-year cohort		5-year cohort	
		Random forest	Logistics regression	Random forest	Logistics regression
Train set	Specificity	0.91	0.657	0.75	0.721
	Sensitivity	0.833	0.792	0.688	0.672
	AUC (95% CI)	0.933 (0.876–0.989)	0.709 (0.615–0.804)	0.774 (0.693–0.854)	0.74 (0.656–0.825)
	PPV	0.526	0.216	0.721	0.694
	NPV	0.977	0.964	0.718	0.70
	F1 score	0.645	0.339	0.704	0.683
Test set	Specificity	0.80	0.52	0.882	0.882
	Sensitivity	0.833	0.833	0.75	0.688
	AUC (95% CI)	0.81 (0.64–0.98)	0.653 (0.363–0.944)	0.829 (0.676–0.982)	0.752 (0.575–0.929)
	PPV	0.333	0.172	0.857	0.846
	NPV	0.976	0.963	0.789	0.75
	F1 score	0.476	0.285	0.80	0.759

**Fig. 6:**
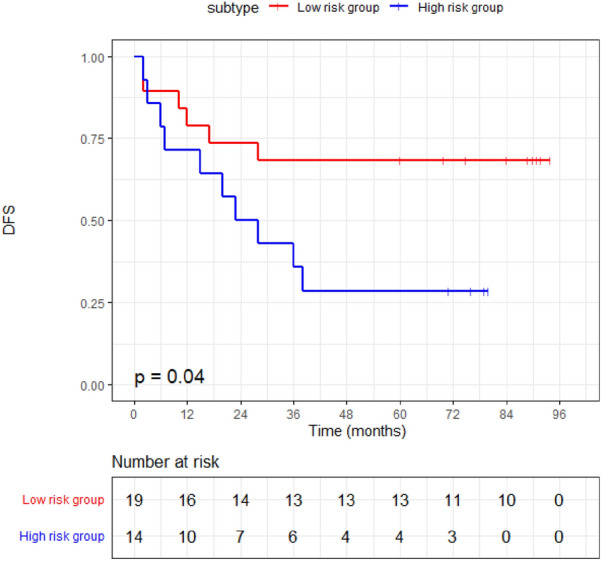
The DFS curve for both the low risk group and high risk group predicted by the model

Finally, in the 5-year cohort test set, we categorized patients into the low risk group if the probability of no event occurring exceeds 50%, and into the high risk group otherwise. Afterwards, we proceeded to plot survival curves for both groups and observed a clear distinction: the low-risk group exhibited significantly longer survival times in comparison to the high-risk group (Fig. 6, *P* = 0.04).

### Model validation

**Fig. 7:**
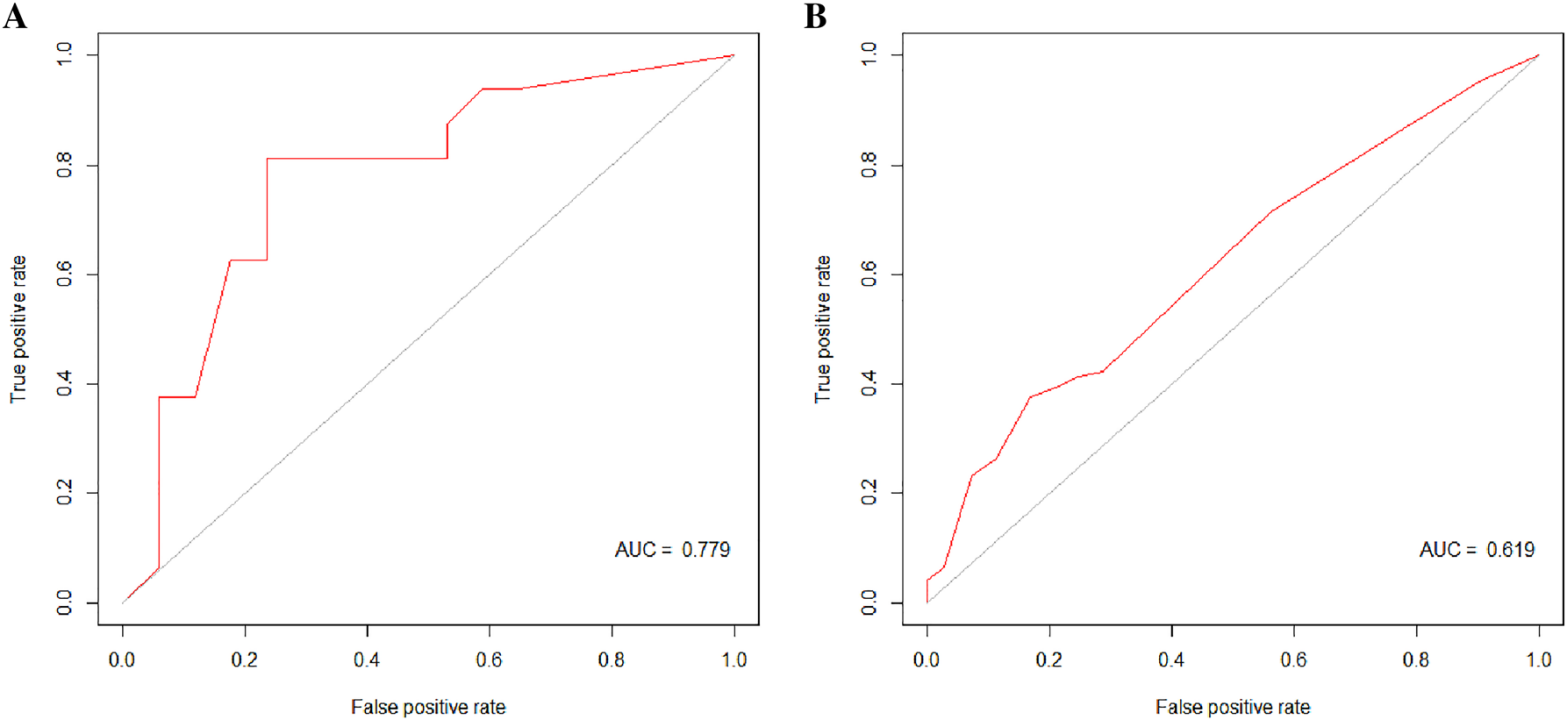
The ROC curve of the random forest model: external validation set of the 5-year cohort (**A**). The ROC curve of the logistic regression: external validation set of the 5-year cohort (**B**)

Totally, 514 patients were identified as no response to NAC from the SEER database, Table 4 shows their clinical features. Out of them, 250 patients had events in the follow-up period. Of the RF model, the specificity, sensitivity, and AUC were 0.765 (95% CI was 0.708–0.814), 0.812 (95% CI was 0.757–0.857), and 0.779 (95% CI was 0.613–0.945), Fig. 7A. The PPV, NPV and F1 score were 0.766, 0.811 and 0.788, respectively. Of the logistics regression model, the specificity, sensitivity, and AUC were 0.833 (95% CI was 0.782–0.875), 0.376 (95% CI was 0.316–0.440), and 0.619 (95% CI was 0.572–0.666), Fig. 7B. The PPV, NPV and F1 score were 0.681, 0.585 and 0.474, respectively. Table 5 presents the relevant information.

**Table 4:** Clinical features of the included SEER data

	Event	Non-event	P-value
Age			0.01
≤40	21	35	
40–60	117	143	
>60	112	86	
cT			< 0.01
T1+T2	113	172	
T3	137	92	
cN			< 0.01
N0+N1	139	203	
N2+N3	111	61	
HR			< 0.01
Negative	91	43	
Positive	159	221	
HER2			0.032
Negative	219	213	
Positive	31	51

**Table 5:** The specificity, sensitivity, AUC, PPV, NPV and F1 score of the validate set in 5-year cohort, respectively

		5-year cohort	
		Random forest	Logistics regression
Validate set	Specificity	0.765 (0.708–0.814)	0.833 (0.782–0.875)
	Sensitivity	0.812 (0.757–0.857)	0.376 (0.316–0.440)
	AUC (95% CI)	0.779 (0.613–0.945)	0.619 (0.572–0.666)
	PPV	0.766	0.681
	NPV	0.811	0.585
	F1 score	0.788	0.484

### Comparison between the RF models and the logistic regressions

The performance of the RF model and the logistic regression model were compared in the 1 year and the 5-year group. The AUC of the RF model was higher than that of logistic regression in the 1-year cohort, the 5-year cohort, as well as in the external validation set. The RF model can reach a satisfactory performance.

## Discussion

In this study, we attempted to develop a machine learning model based on an RF algorithm for predicting the occurrence of events among BC patients. And we identified that compared to the logistic regression, the RF model could reach a satisfactory performance to predict the events occurring of BC patients post-treatment.

RF was first introduced in the early 2000 s. Based on Breiman’s “bagging” idea and the random selection of features, RF could obtain higher accuracy. Cutler has concluded that compared to other classifiers, RF has the advantage of (1) high classification accuracy; (2) identifying variable importance; (3) the ability to perform multiple types of data analysis; (4) modeling complex interactions between explanatory variables [29]. Currently, RF is a common ensemble learning method for classification, regression, and other tasks. In the study by Liu et al. the sensitivity, specificity, and AUC of the random forest were 91.8%, 51.2%, and 0.897 [21]. Wang et al. have established an RF model for recognizing macrosomia. The sensitivity, specificity, and AUC were 91.7%, 91.7%, and 0.953, which was significantly higher than the logistic regression [30]. Furthermore, Huang et al. have developed 9 models based on machine learning to predict prognosis in early elderly triple negative breast cancer, and concluded that these models performed well [10].

Based on previous studies, we retrospectively analyzed the data of BC patients in our hospital and selected 9 variables for comparison among whether patients had an event or not. Then, we included all of these 9 variables for establishing the model. However, the model did not perform very well, which was with a relatively higher OOB error rate and a lower AUC. The importance analysis also showed that only HER2 was significant in the model and almost half of these variables were negatively associated with the outcome variable. Hence, we tried to decrease the number of explanatory variables. After conducting multiple comparisons of variable combinations, we ultimately discovered that in the 1-year cohort, the model exhibited lower error rates and higher performance when utilizing 5 variables. Thus, we used Stage T, stage N, HR, HER2, and P53 as explanatory variables to predict the event. Similarly, in the 5-year cohort, we found that when the number of variables was 3, the model would do better, and we used cT, HR, and HER2 as explanatory variables to predict the even.

For machine learning, interpretability of models is a somewhat subjective quality that cannot be formally defined through rigorous mathematical expressions. In our view, as a general rule, there tends to be a positive correlation between a model's complexity and its accuracy, with greater complexity often leading to reduced interpretability. Indeed, in our study, we couldn’t explain why, in the random forest model, selecting these specific independent variables leads to better predictive performance. However, we cannot abandon the model solely due to its low interpretability.

There were limitations in our work. First, only a part of the bio-data was selected for analysis. Other information such as blood analysis data, CT or MRI imaging data, and pathological imaging data were omitted. Second, we only developed 2 kinds of machine learning models. Third, although we have obtained some valuable conclusions, there were no new findings in Cox regression. Fourth, in the first year, there were only 30 patients occurring event, The data imbalance might indeed affect the model development. The most importantly, we used statistical methods to reduce the representative features for training the random forest model. These might affect the performance of the model. However, through this study, we have demonstrated that machine learning models can achieve promising results in accurately predicting events occurring based on simple clinical variables. In the future, it is of vital importance to supplement the data, such as imaging and pathological images. To extract image variables using deep learning and then construct a model based on these data.

Nevertheless, this study confirmed the effectiveness of the RF model in predicting events in breast cancer patients.. In the future, the study should focus on multi-center research, supplementing the database, especially the imaging and pathological pictures. And improving the variable selecting method. We hope that our work can help clinical decision-making among patients at high risk for recurrence, timely warning, and timely intervention. Further research should include more patients in multiple clinical centers, select more explanatory variables, and base on multiple machine learning and deep learning algorithms to construct predicted models (Additional file 1).

## Conclusion

Given the numerous new BC patients each year, and the great demand to ensure early detection of tumor recurrence, this manuscript discussed the possibility of timely predicting deterioration in BC patients.

### Supplementary Information

**Additional file 1: Figure S1.** The ROC curve of the random forest model: entire patients of the 1-year cohort (A) and 5-year cohort (C). The ROC curve of the logistic regression: entire patients of the 1-year cohort (B).and 5-year cohort (D).

## Data Availability

The data are available from the corresponding author upon reasonable request.

## References

[1] Sung H, Ferlay J, Siegel RL, Laversanne M, Soerjomataram I, Jemal A (2021). Global cancer statistics 2020: GLOBOCAN estimates of incidence and mortality worldwide for 36 cancers in 185 countries. CA Cancer J Clin.

[2] Waks AG, Winer EP (2019). Breast cancer treatment: a review. JAMA.

[3] Early Breast Cancer Trialists' Collaborative Group (EBCTCG). Long-term outcomes for neoadjuvant versus adjuvant chemotherapy in early breast cancer: meta-analysis of individual patient data from ten randomised trials. Lancet Oncol. 2018;19(1):27–39. 10.1016/S1470-2045(17)30777-5 PMC5757427 29242041

[4] Cortazar P, Zhang L, Untch M, Mehta K, Costantino JP, Wolmark N (2014). Pathological complete response and long-term clinical benefit in breast cancer: the CTNeoBC pooled analysis. Lancet.

[5] Spring L, Greenup R, Niemierko A, Schapira L, Haddad S, Jimenez R (2017). Pathologic complete response after neoadjuvant chemotherapy and long-term outcomes among young women with breast cancer. J Natl Compr Canc Netw.

[6] Hou Y, Peng Y, Li Z (2022). Update on prognostic and predictive biomarkers of breast cancer. Semin Diagn Pathol.

[7] Tarighati E, Keivan H, Mahani H (2023). A review of prognostic and predictive biomarkers in breast cancer. Clin Exp Med.

[8] Kos Z, Dabbs DJ (2016). Biomarker assessment and molecular testing for prognostication in breast cancer. Histopathology.

[9] Yau C, Osdoit M, van der Noordaa M, Shad S, Wei J, de Croze D (2022). Residual cancer burden after neoadjuvant chemotherapy and long-term survival outcomes in breast cancer: a multicentre pooled analysis of 5161 patients. Lancet Oncol.

[10] Huang K, Zhang J, Yu Y, Lin Y, Song C (2022). The impact of chemotherapy and survival prediction by machine learning in early elderly triple negative breast cancer (eTNBC): a population based study from the SEER database. BMC Geriatr.

[11] Zheng X, Yao Z, Huang Y, Yu Y, Wang Y, Liu Y (2020). Deep learning radiomics can predict axillary lymph node status in early-stage breast cancer. Nat Commun.

[12] Li C, Liu M, Li J, Wang W, Feng C, Cai Y (2022). Machine learning predicts the prognosis of breast cancer patients with initial bone metastases. Front Public Health.

[13] Asare EA, Liu L, Hess KR, Gordon EJ, Paruch JL, Palis B (2016). Development of a model to predict breast cancer survival using data from the national cancer data base. Surgery.

[14] de Glas NA, Bastiaannet E, Engels CC, de Craen AJ, Putter H, van de Velde CJ (2016). Validity of the online PREDICT tool in older patients with breast cancer: a population-based study. Br J Cancer.

[15] Kindts I, Laenen A, Peeters S, Janssen H, Depuydt T, Nevelsteen I (2016). Validation of the web-based IBTR! 2.0 nomogram to predict for ipsilateral breast tumor recurrence after breast-conserving therapy. Int J Radiat Oncol Biol Phys.

[16] Phung MT, Tin Tin S, Elwood JM (2019). Prognostic models for breast cancer: a systematic review. BMC Cancer.

[17] Yu Y, Tan Y, Xie C, Hu Q, Ouyang J, Chen Y (2020). Development and validation of a preoperative magnetic resonance imaging radiomics-based signature to predict axillary lymph node metastasis and disease-free survival in patients with early-stage breast cancer. JAMA Netw Open.

[18] Massafra R, Comes MC, Bove S, Didonna V, Diotaiuti S, Giotta F (2022). A machine learning ensemble approach for 5- and 10-year breast cancer invasive disease event classification. PLoS ONE.

[19] Mikhailova V, Anbarjafari G (2022). Comparative analysis of classification algorithms on the breast cancer recurrence using machine learning. Med Biol Eng Comput.

[20] Song Y, Yin Z, Zhang C, Hao S, Li H, Wang S (2022). Random forest classifier improving phenylketonuria screening performance in two Chinese populations. Front Mol Biosci.

[21] Liu YH, Jin J, Liu YJ (2022). Machine learning-based random forest for predicting decreased quality of life in thyroid cancer patients after thyroidectomy. Support Care Cancer.

[22] Eisenhauer EA, Therasse P, Bogaerts J, Schwartz LH, Sargent D, Ford R (2009). New response evaluation criteria in solid tumours: revised RECIST guideline (version 1.1). Eur J Cancer.

[23] Schwartz LH, Litière S, de Vries E, Ford R, Gwyther S, Mandrekar S (2016). RECIST 1.1-Update and clarification: from the RECIST committee. Eur J Cancer.

[24] Huang X, Yin YM (2018). Updates of Chinese society of clinical oncology (CSCO) guideline for breast cancer in 2018. Zhonghua Yi Xue Za Zhi.

[25] Gradishar WJ, Moran MS, Abraham J, Aft R, Agnese D, Allison KH (2022). Breast cancer, version 3.2022, NCCN clinical practice guidelines in oncology. J Natl Compr Canc Netw.

[26] Li JB, Jiang ZF (2021). Chinese society of clinical oncology breast cancer guideline version 2021: updates and interpretations. Zhonghua Yi Xue Za Zhi.

[27] Abubakar M, Guo C, Koka H, Sung H, Shao N, Guida J (2019). Clinicopathological and epidemiological significance of breast cancer subtype reclassification based on p53 immunohistochemical expression. NPJ Breast Cancer.

[28] Yaghoobi V, Martinez-Morilla S, Liu Y, Charette L, Rimm DL, Harigopal M (2020). Advances in quantitative immunohistochemistry and their contribution to breast cancer. Expert Rev Mol Diagn.

[29] Cutler DR, Edwards TC, Beard KH, Cutler A, Hess KT, Gibson J (2007). Random forests for classification in ecology. Ecology.

[30] Wang F, Wang Y, Ji X, Wang Z (2022). Effective macrosomia prediction using random forest algorithm. Int J Environ Res Public Health.

